# The Endoscopic Removal of a Detached Dental Implant Cap in the Maxillary Sinus During the Waiting Period: A Case Report

**DOI:** 10.7759/cureus.63243

**Published:** 2024-06-26

**Authors:** Katsuhiko Amano, Chiaki Nishizawa, Yohei Maeda, Susumu Tanaka, Seiji Iida

**Affiliations:** 1 Department of Oral and Maxillofacial Reconstructive Surgery, Graduate School of Medicine, Dentistry and Pharmaceutical Sciences, Okayama University, Okayama, JPN; 2 Department of Oral and Maxillofacial Surgery, Osaka University, Graduate School of Dentistry, Suita, JPN; 3 Department of Otolaryngology-Head and Neck Surgery, Japan Community Healthcare Organization (JCHO) Osaka Hospital, Osaka, JPN

**Keywords:** waiting period, endoscopic approach, missing implant cap, foreign body, maxillary sinus, displacement of dental implants

## Abstract

Displacement of dental implants into the maxillary sinus is one of the common dental complications. However, it is rare that dental implants cause the displacement of multiple components due to separation. Here, we describe an unusual case of a 43-year-old man who had a missing implant in the maxillary sinus after an implant procedure. There was a two-week waiting period before we performed the removal during which the cap had unexpectedly separated from the body and freely moved into the ostium by sinus activity. The body was independently extracted intraorally. The remaining cap was secondly removed by utilizing nasal endoscopy. There were no complications in the postoperative period and the patient reported no symptoms of sinusitis after two months of follow-up. We experience unexpected events in the course of treating a displaced implant into the maxillary sinus. Our case report may provide several learning points for the removal of a missing implant.

## Introduction

A dental implant is an option as a prosthesis in dental treatment. Recent advances in both techniques and materials have improved implant surgery to make it easier, safer, and more common. However, there are several potential complications in the operation, including displacement of dental implants into the maxillary sinus [1,2]. Such an accident could lead to nasal bleeding, continuous oral sinus fistula, and infectious sinusitis. This possibility should be taken into consideration in order to prevent it. Unfortunately, when it does occur, removal of the mislocated implant surgically or endoscopically is required [3-5]. Alternatively, there are some reports that migrated dental implants were naturally discharged into the nasal cavity. However, it took about 8 to 16 months until the foreign bodies were discharged [6].

Performing routine dental treatment and/or oral surgery is always associated with some risk of causing foreign bodies into the oral cavity and surrounding tissues. In addition to displaced implants, other possibilities are over-fillings of root canal materials into sinuses or jaw bones, broken bars when drilling, broken screws and plates in jaw fractures, orthognathic surgeries, etc. [3-8]. The features of foreign bodies might influence the technical difficulties in those removal. For instance, removing a single large solid body may be easier, while multiple smaller and fragile pieces will be harder to remove completely. Dental implants consist of several parts, so not all cases involve a single missing solid implant body.

In this report, we report our clinical experiment with dental implant migration into the maxillary sinus and the oral and endoscopic approaches for the removal.

## Case presentation

**Figure 1:**
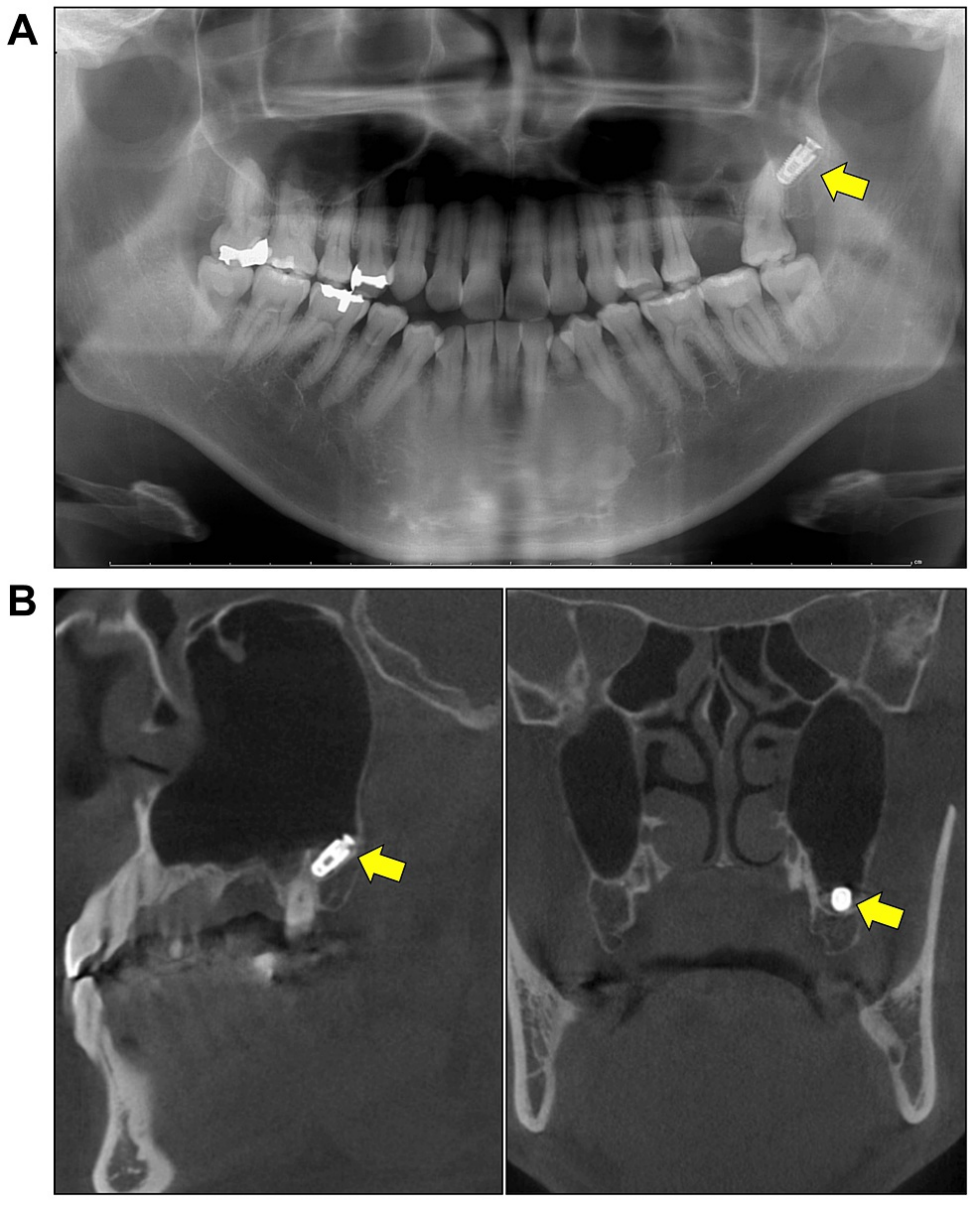
Displacement of a dental implant into the maxillary sinus (A) Panoramic radiograph and (B) cone-beam computed tomography (CBCT) sagittal image (left) and coronal image (right) show the dislocation of the dental implant into the posterior part of the left maxillary sinus floor which was posterior to the broken upper left first molar region. The yellow arrows mark the implant.

A 43-year-old man, a non-smoker and without any systemic diseases, visited our department in September 2020 after receiving an implant procedure at a nearby dental clinic. The implant surgery was for his missing left upper first molar, and the operating dentist noticed that the implant had disappeared when strengthening the implant cap over the inserted implant body. He was then introduced to our department for diagnosis and treatment for the missing implant. The initial clinical screening indicated that the patient did not have active bleeding, severe pain or purulent discharge from his oral wound and nose. We utilized a panoramic radiograph and cone-beam computed tomography (CBCT) to find it and the dislocated dental implant was recognized in the posterior part of the left maxillary sinus floor in the broken upper left first molar region (Figures 1A-1B). Based on the consultation with the patient, we did not take immediate action but scheduled an appointment to plan the removal surgery under intravenous sedation in two weeks.

**Figure 2:**
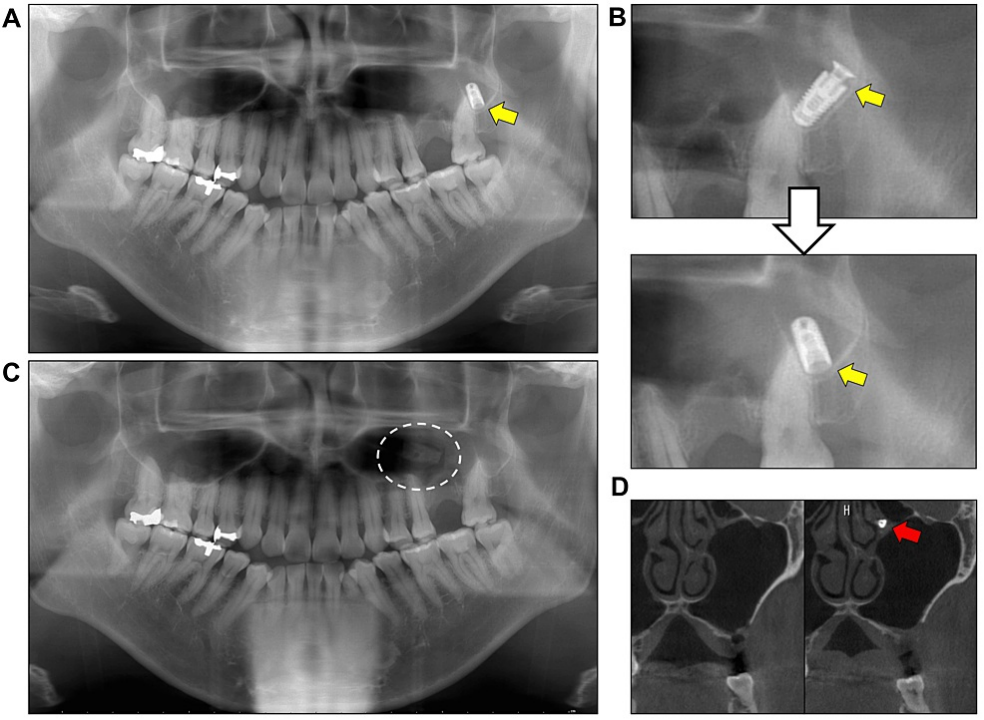
Separated implant cap found in the natural ostium (A) Immediately preoperative radiographic image. (B) Comparison of the implant position in the X-ray images between initial screening (upper) and preoperative data (lower). Note that the implant moved slightly in the sinus during a waiting period. Also, note the implant structure with a missing cap. (C) The postoperative radiographic image confirmed the successful removal of the implant body. (D) Coronal CBCT images. The left panel is an image at initial screening and the right panel is one after the intra-oral antrostomy. Note the separated cap in the left natural ostium of the middle nasal meatus. The yellow arrows indicate the displaced implant. The white circle shows the region of intra-oral antrostomy. The red arrow marks the missing cap.

Two weeks after the initial contact we removed the displaced implant in the sinus by classical open surgery through the canine fossa under intravenous sedation. A preoperative panoramic radiograph indicated that the implant in the sinus had moved slightly during the waiting period but did not markedly change position (Figure 2A). However, the healing cap could not be found while the implant body was being extracted (Figure 2B). A postoperative radiographic image confirmed the expected removal of the implant body (Figure 2C). Notably, further CBCT examination found that the missing healing cap was in the left natural ostium of the middle nasal meatus (Figure 2D). It is assumed that the cap was separated from the implant fixture and independently moved through the maxillary sinus activity into the ostium during the waiting period. The operation time was 58 minutes and blood loss was limited. We used midazolam and 1% propofol for sedation. After the treatment, the first removal operation and the displaced remaining cap were explained to the patient. Based on consultation with the patient, we decided to introduce him to otorhinolaryngologists to consider endoscopic removal of the dental implant cap.

**Figure 3:**
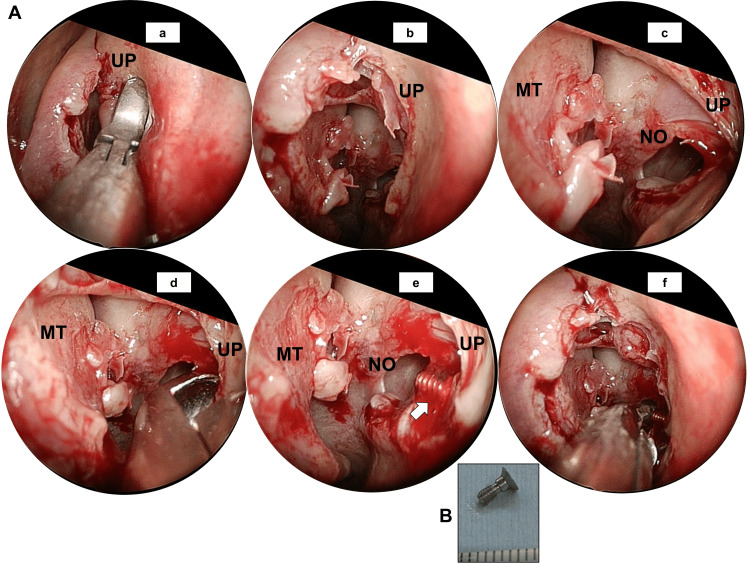
Endoscopic removal of the remaining implant cap (A) a: The bottom part of the uncinate process was removed by forceps. b: Findings with 0-degree endoscopy after removal of the bottom part of the uncinate process. c: Findings with 70-degree endoscopy after removal of the bottom part of the uncinate process. The left natural ostium of the maxillary sinus was identified. d: The remaining uncinate process was removed laterally. e: The missing cap was clearly visible (indicated by a white arrow). f: The missing cap had been caught by forceps. (B) The missing cap was finally removed. UP: uncinate process of the ethmoid; NO: natural ostium; MT: middle turbinate

The endoscopic operation was performed a month later as illustrated in Figure 3A. Briefly, the endoscope was inserted from the left nostril to the middle nasal passage under local anaesthesia. We observed chronic inflammatory mucosa on the uncinate process of the ethmoid and removed the mucosa using endoscopic forceps (Figures 3Aa-3Ab). We then identified the left natural ostium of the maxillary sinus (Figure 3Ac). We further extended the surgical field laterally, removing the remaining uncinate process (Figures 3Ad-3Ae). The cap was finally successfully removed by this method (Figures 3Af-3B). The postoperative period was uncomplicated and the patient reported no symptoms of sinusitis after two months of follow-up.

## Discussion

Unlike a simple procedure for foreign body removal, our case report includes some unusual events that occurred during treatment. First, the implant body and cap were unexpectedly separated during the waiting period for surgery. A dental implant consists of several parts and it is possible to have multiple missing pieces after the dislocation. In our case, the original dentist caused an accidental insertion when strengthening the cap. Therefore, the connection between the missing implant body and cap might not have been completely tight and some loosening had occurred. Thus, the possibility that combined foreign bodies could dissociate in the tissue should be taken into consideration when planning and performing the removal. Moreover, our case indicates that it is important to exercise caution and keep the waiting time as short as possible even in the absence of symptoms [9]. However, there are some reports that foreign bodies in the sinuses can be naturally discharged into the nasal cavity [6,7,9-12]. In this case, the migrated implant cap was very small and found right next to the natural nasal ostium in the CBCT image. Thus, it could have been naturally discharged into the nasal cavity at some point in the future. But this is difficult to predict and we retrospectively noticed that the endoscopic approach was a better way to take since chronic inflammation and swelling of nasal mucous membrane surrounding the missing implant cap were observed. These reactions might have prevented that natural discharge. Hence, it is a case-by-case determination to “wait and see” that depends on the size and location of the foreign bodies [6]. Patients should obviously be informed of any risks of removal surgery versus waiting for a possible natural discharge [6].

The second point to discuss is that the dental implant cap independently moved into the natural ostium of the middle nasal meatus which is not visible in an immediate preoperative panoramic radiograph. As shown in Figure 2B, the detachment of the cap from the body could have been assumed and we in fact did not find it during the operation using the conventional approach. Reassessment using CBCT located the missing cap at the natural ostium. Accordingly, careful attention should be paid when examining foreign bodies in the sinuses and the nose using a panoramic radiograph since it does not cover the ostium of the middle nasal meatus. In addition, reassessment of the condition and position of foreign bodies are necessary when there is a waiting period before the removal operation [9].

Finally, our case illustrates that collaboration between dentists and otolaryngologists is valuable and may be required depending on the placement of a foreign body. There are various methods for removing a foreign body depending on the size and position. Common techniques for sinuses are an external approach by oral antrostomy, a combined approach of intranasal and oral antrostomy, or endoscopic surgery [1-5,8,13-15]. In our case, the implant was found near the posterior part of the left maxillary sinus floor and we performed a direct procedure that allowed a better visualization of the surgical area. However, since there was a waiting period of about two weeks, a detachment of the cap from the implant body occurred. The cap moved into the left cave of the middle nasal meatus and granulation tissue covered the cap, preventing us from finding it in the oral antrostomy. Hence, we requested otolaryngologists to perform a nasal endoscopy. We subsequently researched previous papers associated with dental foreign bodies in the nasal cavity and found 10 previous cases (Table 1) [6-15].

**Table 1:** Dental foreign bodies found in the nasal cavity

Case no.	Authors	Year of publication	age	Foreign body	Removal procedure
1	Higo and Hukushima et al. [10]	1994	n.d.	Gutta-percha filling	Natural discharge
2	Yokomizo et al. [11]	2007	50	Dental implant	Natural discharge
3	Akashi et al. [7]	2008	25	Dental cutting bar	Natural discharge
4	Abe et al. [13]	2011	68	Dental-impression silicone	Endoscopic surgery
5	van de Loo et al. [9]	2013	65	Dental implant	Natural discharge
6	Tachibana et al. [6]	2013	52	Root canal filling	Natural discharge
7	Ueda et al. [12]	2017	64	Dental implant	Natural discharge
8	Menezes et al. [14]	2019	n.d.	Dental implant	Endoscopic surgery
9	Li et al. [15]	2020	23	Dental implant	Endoscopic surgery
10	Du et al. [8]	2021	55	Fractured instrument	Endoscopic surgery

## Conclusions

Our case report suggests that it is indispensable for clinicians to take appropriate images and accurately evaluate clinical data when removing foreign bodies from the maxillary sinus. In addition, it is essential to consider the possibility for foreign bodies to dissociate in the maxillary sinus by sinus activity, especially if there is a waiting period. We believe that our case report could provide several learning points that demonstrate unusual events that may occur during the removal procedures for a missing implant.
